# Colloidal crystals with diamond symmetry at optical lengthscales

**DOI:** 10.1038/ncomms14173

**Published:** 2017-02-13

**Authors:** Yifan Wang, Ian C. Jenkins, James T. McGinley, Talid Sinno, John C. Crocker

**Affiliations:** 1 Department of Chemical and Biomolecular Engineering, University of Pennsylvania, 220 S. 33rd Street, Philadelphia, Pennsylvania 19104, USA

## Abstract

Future optical materials promise to do for photonics what semiconductors did for electronics, but the challenge has long been in creating the structure they require—a regular, three-dimensional array of transparent microspheres arranged like the atoms in a diamond crystal. Here we demonstrate a simple approach for spontaneously growing double-diamond (or B32) crystals that contain a suitable diamond structure, using DNA to direct the self-assembly process. While diamond symmetry crystals have been grown from much smaller nanoparticles, none of those previous methods suffice for the larger particles needed for photonic applications, whose size must be comparable to the wavelength of visible light. Intriguingly, the crystals we observe do not readily form in previously validated simulations; nor have they been predicted theoretically. This finding suggests that other unexpected microstructures may be accessible using this approach and bodes well for future efforts to inexpensively mass-produce metamaterials for an array of photonic applications.

## Abstract (web summary)

Colloidal crystals arranged in a diamond lattice are desirable for photonic applications, yet are challenging to create. Here, Wang *et al*. show the self-assembly of a binary system composed of two interlocked diamond structures with lattice spacing comparable to the wavelength of visible light.

Metamaterials, typically consisting of optical wavelength-sized building blocks arranged in periodic arrays, promise the creation of unique photonic technologies^1^. A particularly favourable three-dimensional metamaterial consists of transparent spheres arranged on a cubic diamond lattice^2^, which has led to a multi-decade effort to form diamond structures using lithography^3^, micromanipulation^4^ or holography^5^ as well as self-assembly approaches based upon liquid crystals^6^, nanoparticles^7^,^8^,^9^ or colloidal crystallization^10^,^11^,^12^,^13^,^14^,^15^. The notorious difficulty of forming a diamond lattice using colloidal crystallization is due to the structure's low filling fraction and mechanical instability; colloids with short-ranged and isotropic attractive interactions will favour a denser and more highly coordinated structure. Different proposed approaches for self-assembling colloidal diamond crystals are summarized in Fig. 1. One approach is to use isotropic interactions that combine a long-range repulsion with a short-ranged attraction^10^,^13^,^14^ (Fig. 1a). While this approach has led to the experimental formation of diamond-like crystals of oppositely charged nanoparticles^7^, it does not appear to be adaptable to the larger scales required for photonic materials. A second proposed approach uses ‘patchy colloids' that only interact through small patches on their surfaces^16^ (Fig. 1b) to mimic the tetrahedral directional interactions of carbon atoms in a diamond lattice, but is challenging due to competition with a thermodynamically preferred amorphous tetrahedral liquid or gel^17^,^18^,^19^,^20^. A third approach is to form a denser and more highly coordinated structure that contains a diamond lattice of one compositionally distinct species, which has a second lattice or ‘scaffold' of another species in its interstitial space, which prevents the diamond lattice from collapsing or rearranging. One example is isomorphic to the MgCu_2_ Laves phase^12^,^21^ (Fig. 1c), in which the ‘scaffold' consists of smaller ‘Cu' spheres^11^ arranged into a second diamond lattice of tetrahedral clusters of spheres^15^ (also known as the pyrochlore lattice).

Here we demonstrate a simple self-assembly method for growing ‘scaffolded' diamond crystallites from roughly 400 nm diameter polymer microspheres, with a lattice spacing comparable to that of visible light. First, we prepare two slightly different-sized species of microspheres with complementary DNA strands grafted to their surfaces^22^, which form molecular bridges^23^,^24^,^25^ between them when they come within ∼30 nm of contact. Under conditions where the DNA bridge formation is rapid and reversible^26^, the spheres experience a short-ranged attraction that drives the spontaneous nucleation and growth of large colloidal crystals^23^,^27^,^28^,^29^,^30^,^31^. Many of the resulting crystals have a well-ordered ‘double diamond' (DD) or B32 structure—where the ‘scaffold' is simply a second diamond lattice of smaller and different-composition spheres (Fig. 1d) interpenetrating the first. This structure is isomorphic to the NaTl Zintl phase^32^ in atomic solids. Our observation of such a DD or B32 lattice has not been predicted for this system and is completely unexpected. While this structure has been reported once in a nanoparticle system^8^, its thermodynamic stability requires next-nearest neighbour interactions^32^,^33^ that are not present with DNA colloids^32^,^33^. Indeed, simulations show that the binding energy of our DD crystallites is smaller than for co-occurring crystallites having a CsCl structure. Moreover, matched simulations fail to nucleate or grow such DD crystals directly from a fluid phase, suggesting non-classical mechanisms for both processes. This explanation is supported by the crystallites extreme structural deformability and the experimental observation of reconstructed surfaces. Crosslinking^34^ such crystals and dissolving the smaller scaffold species could provide a facile and scalable route for self-assembling diamond crystals that would have interesting and useful metamaterial properties.

## Results

### Formation and imaging of binary colloidal crystals

To form DD crystals, we use an aqueous suspension containing two types of similarly sized microspheres with diameters of roughly 400 nm, which have been engineered to have controllable and chemically specific interactions. For the two species ‘A' and ‘B', our four adjustable parameters are two ‘like' attraction strengths, *U*_AA_ and *U*_BB_, one ‘unlike' one, *U*_AB_=*U*_BA_, (defined as positive-valued parameters) and the spheres' diameter ratio, *σ*_A_/*σ*_B_. The interactions are realized and modulated by grafting various amounts of complementary DNA strands to the two particle species' surfaces^22^,^23^, see Methods for details. When two microspheres bearing complementary DNA sequences come near contact, DNA hybridization leads to molecular bridges that pull the spheres together. At sufficiently high DNA density^26^,^31^ the attraction resembles an isotropic, reversible interaction potential^24^,^25^ with a range of about 30 nm. All particle pairs also exhibit a soft repulsion near contact due to the compression of their DNA brushes^17^, having a range of roughly 10 nm. Since both length scales are much shorter than a particle radius, we consider the particles to act nearly as ‘sticky' hard spheres, whose packing is determined by the size of their hard cores, but with an additional energy benefit when spheres with complementary DNA are in contact. Temperature provides a convenient means to modulate the colloidal interactions, since DNA bridges dissociate at elevated temperature. To form crystals, we place a binary suspension of 20% total particle volume fraction in a slowly cooling hot water bath. As the temperature falls, the attractive interactions become gradually stronger until crystals form by homogeneous nucleation.

The resulting, typically polyhedral^29^ crystallites are permanently crosslinked by enzymatic ligation^34^, mounted in a high-index mounting medium and imaged on a confocal microscope. The two particle types are stained with different dyes: smaller A spheres in green, and larger B spheres in red, which can be imaged separately. With the particle sizes and mounting medium we use, the crystallites are effectively transparent, and we can observe both their global shape and lattice structure throughout their depth. While confocal microscopy cannot resolve the three-dimensional structure of sub-micron lattices in the depth direction, it can resolve the particle arrangement and spacing in two-dimensional focal plane slices. The resulting images resemble the superposition of two adjacent crystal planes parallel to the focal plane. By matching these patterns to computer-rendered lattice models from different viewing angles, for an ensemble of crystallites and two colour channels, the three-dimensional lattice structures can be reliably inferred.

### Structure and incidence of DD crystals

Many DD crystallites we observe have the form of a cuboctahedron having square (100) and triangular (111) faces, shown in Fig. 2 and Supplementary Fig. 1, and contain order 10^4^ microspheres. Crystallites sediment and typically come to rest on a flat facet, aligning the focal plane with the faceting lattice directions. The diamond structure is determined from images that show the expected lattice symmetry, orientation and spacing when the focal plane is parallel to (100) and (111) facets. Moreover, the two different particle species consistently show the same lattice symmetry, orientation and spacing, indicating our crystallites consist of two interpenetrating, identical diamond lattices, see Methods. Other DD crystallites are somewhat less regularly shaped and display prominent faces normal to (211) lattice directions. The lattice here resembles a rectangular array of doublets (foreshortened pairs of spheres), that also closely matches computer-generated models for the DD lattice along the (211) direction, in both colour channels, shown in Fig. 3. A larger set of DD crystal micrographs are displayed in Supplementary Figs 2 and 3. A large number of alternative structures were examined to see if they could explain our images (see Methods for listing), none were even able to qualitatively capture the results, let alone do so with the correct lattice spacings.

We find experimentally that the occurrence of DD crystallites requires the particle diameters to differ slightly (*σ*_A_/*σ*_B_=0.96, 0.88 or 0.85, never for *σ*_A_/*σ*_B_=1), have strong unlike interactions, *U*_AB_≫*U*_BB_, weak interactions between the larger spheres, *U*_BB_>0, and show no dependence on those between the smaller spheres, *U*_AA_. The observations regarding the interactions suggest that contacts between the larger spheres are essential to DD crystal formation and stability, while contacts between the smaller spheres are not. Even under the most favourable conditions, however, the majority (>80%) of crystallites formed are isostructural to CsCl, as expected theoretically and reported previously for same-sized spheres^29^,^31^; the incidence of DD crystals under different conditions is summarized in Supplementary Table 1. Reproducibility was excellent; all dozen experiments satisfying the conditions above, across several different particle formulations yielded DD crystals.

The DD crystal has very low nearest-neighbour coordination compared with CsCl (in which every A and B particle has eight AB contacts with neighbours). For an ideal diamond structure of B spheres, each B sphere has only four (weak) B–B contacts; since the A spheres are smaller than the tetrahedral interstice of B's surrounding them, each A can only form at most two (strong) A–B contacts simultaneously in an undistorted DD lattice. Overall, this presents a puzzle: it would appear that the binding energy of DD crystallites due to short-ranged attractions is significantly less than that of CsCl crystals, and yet they both form under similar conditions.

### Simulated DD crystals are deformable

**Figure 1:**
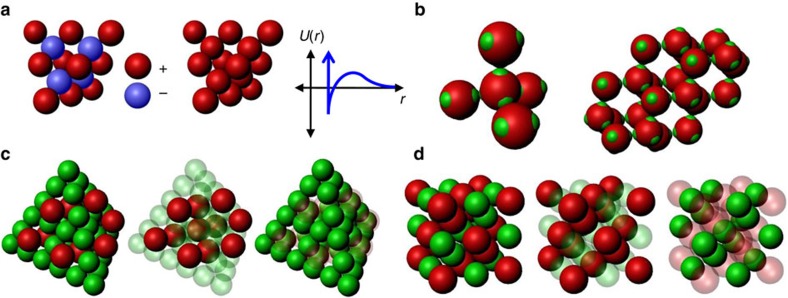
Proposed approaches for making diamond-like colloidal crystals. (**a**) A simple diamond lattice can be stabilized by oppositely charged particles occupying alternating lattice sites, or with a single particle type having a short ranged attraction and long-ranged repulsion. (**b**) Particles that adhere through tetrahedrally arranged patches may form a diamond lattice. (**c**) An MgCu_2_ Laves phase consists of a diamond lattice (red) surrounded by a scaffold of small spheres (green) arranged in tetrahedra. (**d**) Our approach forms a double diamond (DD) (or B32) lattice consisting of two interpenetrating diamond lattices (red and green).

**Figure 2:**
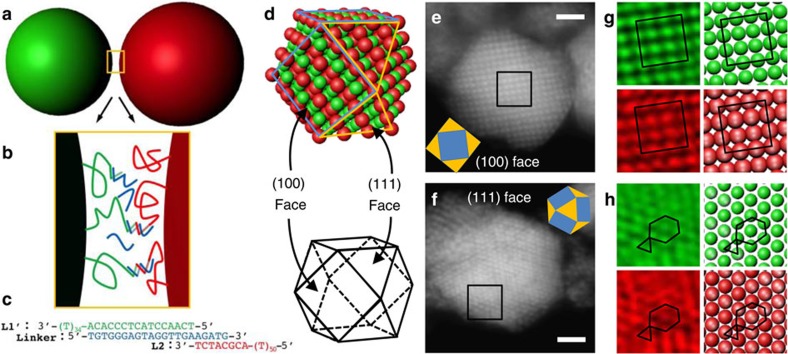
Polyhedral crystallites have a double-diamond structure. (**a**) When DNA-grafted microspheres come near contact, they experience an attractive interaction due to bridges of DNA, (**b**) formed from two grafted single-stranded DNAs (green, red), both hybridized to a linker strand (blue) to form a short double-stranded segment. (**c**) The nucleotide sequence of the strands forms a ligatable ‘nick' between the two grafted strands (green, red). (**d**) The attraction drives the formation of double-diamond (DD) crystallites with cuboctahedral form having six square (100) faces and eight triangular (111) faces. (**e**) Confocal section of a crystallite mid-plane, viewed along the (100) direction shows a square profile (smaller green spheres shown). (**f**) Confocal section of a crystallite mid-plane, viewed along (111) shows an hexagonal profile (smaller green spheres shown). (**g**) Zooming into the boxed section of **e** (left panels) both the small (green) and large (red) particles display square lattices matching an ideal DD crystal model at the same scale (right panels). (**h**) Zooming into the boxed section of **f** (left panels) both the small (green) and large (red) particles display triangular lattices that are slightly distorted relative to the expected ideal DD lattice (right panels). Scale bar is 2 μm. Unprocessed three-dimensional data set available as Supplementary Movie 1.

**Figure 3:**
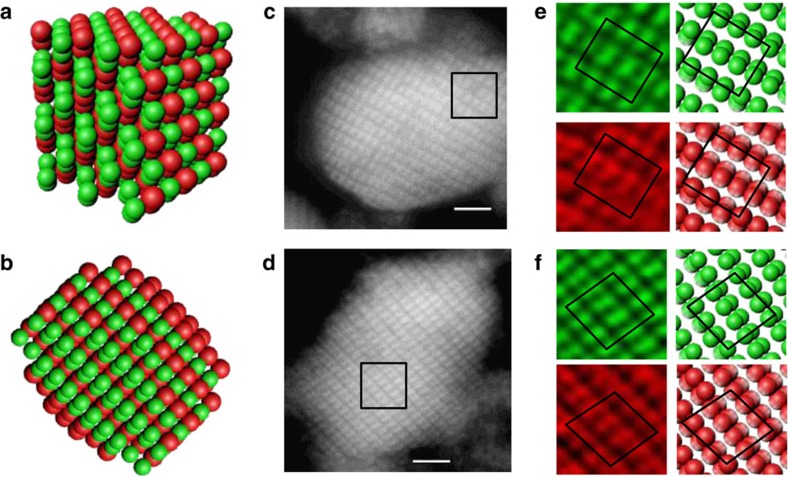
Double-diamond crystallites display diamond (211) structure. Schematic of double-diamond crystallites viewed obliquely, (**a**) and along the (211) lattice direction (**b**) the latter appears as a rectangular lattice of foreshortened pairs of spheres. (**c**,**d**) Confocal sections (green channel, small particles) of the mid-planes of two different, typical crystallites having a (211) viewing orientation. (**e**) Zoom into the boxed region of the crystallite in **c** reveals both the small (green) and large (red) particles display rectangular lattices of doublets (left) resembling an ideal DD crystal rendered at the same scale (right). (**f**) Zoom into the boxed region of the crystallite in **d** reveals both the small (green) and large (red) particles display parallelogram lattices of doublets (left) having a ∼10° shear angle relative to the ideal DD crystal rendered at the same scale (right), indicating a distorted DD lattice. Scale bar is 2 μm. Unprocessed three-dimensional data set available as Supplementary Movie 2.

**Figure 4:**
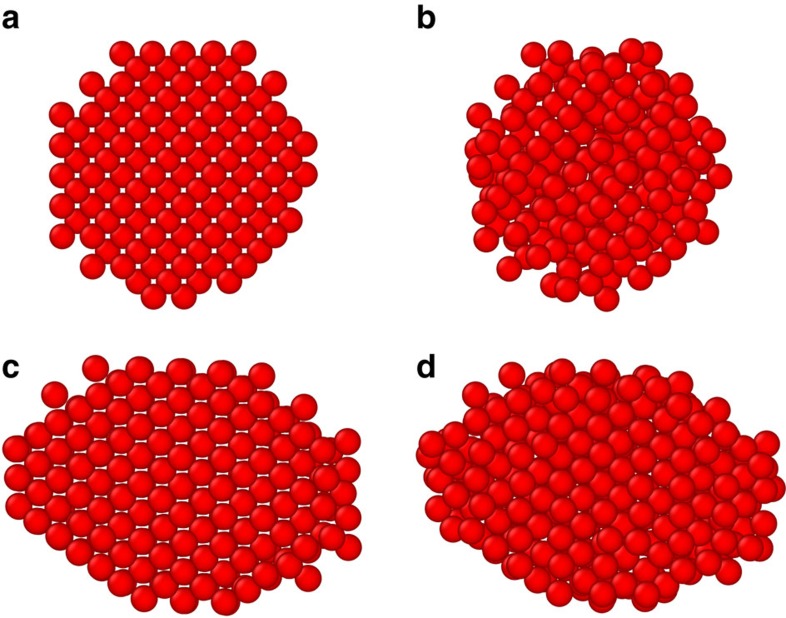
Double-diamond crystals are stable in simulation. (**a**) Rendering of the large, 445 nm diameter particles in an ideal DD lattice (100) orientation, size ratio 0.88. (**b**) Snapshot of the same lattice as **a** after thermalization in a BD simulation, with interactions *U*_BB_=3 *k*_B_*T*, *U*_AB_=6 *k*_B_*T*. (**c**) A lower energy lattice resulting from shearing the ideal DD lattice along a zero-energy mode until it achieves more A–B-type contacts. (**d**) Snapshot of the same lattice as **c** after thermalization.

To understand the occurrence and apparent stability of DD crystals, we performed a series of Brownian dynamics (BD) simulations using realistic and validated DNA interaction potentials^24^, bracketing the range of interaction strengths expected in the experiments, see Methods and Fig. 1. First, we constructed spherical and octahedral crystallites initialized to an ideal DD structure (with the smaller A spheres slightly shifted to form two A–B contacts each), Fig. 4a. For sufficiently strong interactions (*U*_BB_>3 *k*_B_*T*, *U*_AB_>6 *k*_B_*T*) the crystallites were morphologically stable upon thermalization but also exhibited moderate densification that reduced the mean size of the tetrahedral interstices occupied by A particles, and increased the mean number of A–B contacts (from 2 to an average of ∼4.64 in the bulk), see Fig. 4b. This densification is one manifestation of a preponderance of zero-energy or ‘floppy' modes in the DD structure, corresponding to deformations that have zero associated energy penalty because they do not stretch or break favoured sphere–sphere contacts. Inspired by our prior work^29^ in other ‘floppy' crystals of DNA colloids, we evolved the ideal DD crystal along an arbitrarily chosen shear-like floppy mode^35^ until a more highly coordinated lattice was reached (with 4A–B contacts per B particle in the bulk), see Fig. 4c and Methods. On thermalization, this new ‘sheared DD' structure reached a coordination that was still higher (to ∼5.4A–B contacts per B particle in the bulk), Fig. 4d, but which was still far short of that afforded by the CsCl structure (8A–B contacts). Intriguingly, ∼50% of our experimental crystallites show clear lattice distortions (bond angles deviate ±10°, and lattice spacings deviate ±10%), that qualitatively resemble those of the ‘sheared DD' structure (Fig. 4d and Supplementary Table 1). This finding suggests that some of our DD crystals may have transformed, as seen in other floppy crystals^29^,^35^, but the volume of our experimental data is not sufficient to allow meaningful statistical tests of this idea.

### DD crystals do not nucleate in simulation

Given the apparent energetic unfavourability of the DD lattice (with or without densification or transformation), the experimental observations could be explained were the DD crystal kinetically favoured, exhibiting faster nucleation or growth rates than CsCl. However, BD simulations seeded with the above ideal and sheared DD crystallites showed no significant growth for any plausible particle interactions or volume fractions. The volume fractions tested ranged from 1.25 to 40%, with B–B binding strengths ranging from 0 to 20 *k*_B_*T*, A–A binding strengths ranging from 0 to 10 *k*_B_*T* and A–B binding strengths ranging from 0 to 30 *k*_B_*T*. While some A–A contacts were observed in the densified configurations, the stability of the crystallites was essentially independent of the A–A binding strength. Crystallite seeds were generally found to melt for A–B binding strengths below 5 kT.

In addition to attempts at growing DD seeds with direct BD simulations, umbrella sampling simulations also were performed. In these simulations, a bias potential of the form *U*_B_=*k*(*n−n*_T_)^2^ was imposed on the system, where *n* is the total number of particles in a DD seed and *n*_T_ is a target value for the seed size. The bias potential is designed to drive the simulation towards configurations that correspond to the target DD crystallite size and allows for the extraction of the free energy of the crystallite at that size^36^,^37^. The number of particles in the crystallite at any given configuration was determined on the basis of a Steinhardt bond-orientational order parameter^38^; see Methods. Crystallites of various sizes (containing up to 400 particles) in both sheared and unsheared DD configurations were used as initial seeds and allowed to evolve in the umbrella sampling simulations. For all initial configurations and binding energies in the ranges noted above, the equilibrium crystallite size was found to be smaller than the target, suggesting that the crystallites were sub-critical. By comparison, the critical nucleus size for CsCl crystallites with comparable A–B binding energies is an order-of-magnitude smaller—consistent with the fact that CsCl is observed to nucleate and grow spontaneously in simulation. These observations do not conclusively rule out direct homogeneous nucleation of DD: it is possible that the order parameters we considered are not optimally aligned with the growing structure, or that the same kinetic barriers that were operational in the direct growth simulations also prevented proper equilibration in the umbrella sampling runs. Interestingly, previous simulations^8^ of the nanoparticle analogue of our system (which is energetically favourable) also fail to show spontaneous nucleation or growth of the DD structure.

## Discussion

**Figure 5:**
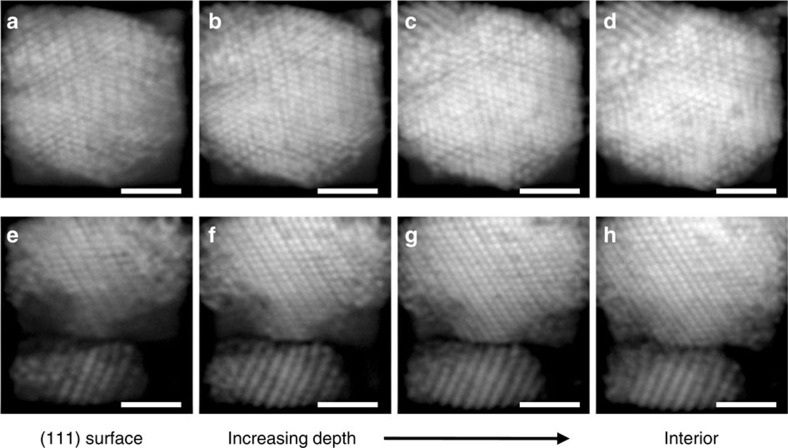
Crystallites surfaces display lattice reconstruction. On the surface of (111) crystallite facets, pairs of smaller, A particles appear to draw together, forming doublets. This gives rise to a banded or striped density modulation. (**a**–**d**) In some crystallites, this banding is incoherent or can exist in multiple directions resulting in small rhombi of four particles, as in **b** upper right. (**e**–**h**) In other crystallites, the banding is more coherent, spanning the crystals' surface and penetrating deeper into the interior. No obvious reconstructions are apparent for (100) or (211) facets. Images show confocal sections separated by 0.5 μm in depth, lightly processed with a digital sharpen filter. Scale bar is 2 μm.

Taken together, the experiments and matched simulations present a conundrum; despite the simulations being broadly successful at capturing the behaviour of these DNA-colloid systems for forming other crystals^28^,^35^,^39^,^40^, they fail to capture the experimental occurrence of DD crystallites. One possibility is that the phase that nucleates and grows initially is not DD, but an unknown ‘parent' phase that transforms to the DD structure once the crystallite has grown to a finite size. Motivating this possibility is the observation of similar Martensitic transformations in other DNA-colloid^29^,^35^ and DNA-nanoparticle^41^ crystallites. This hypothesis would suggest that the nucleating configuration is governed by subtle rearrangements that have relaxed out in the fully grown crystallite, but which must be known to reliably compute nucleation barriers using current methods. Predicting suitable rearrangements *a priori* is made difficult by the extreme floppiness of the DD lattice. For example, in a cubic crystallite with 432 particles, the DD lattice exhibits 631 floppy modes, compared with only 93 for the CsCl lattice we studied previously^35^. An alternative but related explanation is suggested from experiment: the (111) facets in the DD crystallites consistently display clear reconstruction near the surface, resulting in a banded structure, while the crystal deeper inside remains well ordered, as shown in Fig. 5. Presumably, reconstructions such as these allow the formation of additional A–B contacts, reducing the surface-free energy in a similar manner to the well-studied reconstructions in diamond-like atomic systems such as silicon^42^. It seems possible that similar reconstructions in the critical nucleus could lower the nucleation barrier. Notably, the lack of observation of DD crystals when using same-sized A and B spheres, and their maximum occurrence at size ratio ∼0.88 may provide useful clues for future elucidation of the structure of the critical nuclei or transformational intermediates.

Scale-up of our DD crystallites to macroscopic materials would likely benefit from controlled nucleation on a microfabricated template^11^, perhaps along the (100) or (211) growth faces that are nearly flat and appear to display little surface reconstruction. Photonic applications will also require the substitution of high refractive index microspheres as well as the cross-linking, chemical removal of the smaller spheres and freeze- or critical-drying. Beyond such engineering concerns, discovering the relevant nucleation pathway and surface relaxation processes for our observed DD crystals will require further experiments and simulations, but whose resolution may open up currently unanticipated pathways for self-assembling other diamond-like or perhaps even more exotic structures.

## Methods

### DNA sequences

L1′ (ligatable, phosphorylated):

5-/5Phos/TCAACCTACTCCCACATTTTTTTTTTTTTTTTTTTTTTTTTTTTTTTTTT/3AmMO/-3

L2 (complementary via linker to L1 & L1′, non-phosphorylated):

5-/5AmMC6/TTTTTTTTTTTTTTTTTTTTTTTTTTTTTTTTTTTTTTTTTTTTTTTTTTACGCATCT-3

L12_Linker_5 (5 base interaction region+nick site+16 base region):

5- TGTGGGAGT AGGTTGAAGATG-3.

### F108 polymer and DNA conjugation

Unless specified, all fluid handling performed in autoclaved disposable plastic Eppendorf tubes. Glass vials (3 ml), caps and stir bars were washed three times with bio-water, Alconox, Acetone and Ethanol before use, dried on the hot plate at level 4 for 30 min–1 h, cooled with compressed air, and finally cooled to room temperature on the bench. An amount of 500 mg F108, 2 ml dichloromethane and 30 μl TEA were added to the glass vial, allowed to dissolve completely on a heat plate with stir bar mixing, and then 100 mg of fresh 4-NPCF were added and dissolved. The glass vial was then wrapped with parafilm, put on ice and allowed to react for 3–5 h. Four washing solutions were prepared and frozen at −20 °C in clean 50 ml centrifuge tubes, the first contained 14.6 ml ethanol and 0.4 ml HCl, the other three were 14.9 ml ethanol and 0.1 ml HCl. After the reaction, the first washing solution was added, F108 precipitated, the tube was then shaken and chilled at −20 °C for 30 min to complete precipitation, centrifuged at 4,000 r.p.m. for 6 min at 2 °C to form a pellet, and the supernatant discarded. This process was repeated three more times with the remaining washing solutions. After the final wash, the supernatant was discarded and the pellet was warmed by hand until fully redispersed. Activated F108 was split into multiple tubes and dried overnight under vacuum. These samples remain useable for >2 months when stored at −20 °C.

A volume of 15 μl of DNA solution (2,000 μM, in this paper, DNA strands we used were L1′ and L2, see above) was mixed with 1 μl 1 M pH=10 carbonate buffer. An amount of 15 mg activated Pluronic F108 (dried from −20 °C storage) was dissolved in 1 ml 10 mM, pH=4 citric acid buffer, gently vortexed to full dissolution, settled by micro-centrifuge and used immediately. Then, 4 μl F108 in citric acid solution was added to 16 μl DNA buffer solution (total volume 20 μl), gently vortexed for 30 min (after which a yellow reaction product was evident) and settled by micro-centrifuge, then incubated overnight at room temperature. The F108-grafted DNA solution can be stored up to 2 months at 4 °C.

### Particle preparation and DNA grafting

First, 80 μl polystyrene (PS) microspheres/beads were washed three times by dilution with 920 μl bio-water, centrifugation at 8,000 r.p.m. for 35 min, and discarding of supernatant. The pellet was weighed on a micro-balance after the last step to verify that no mass was lost. Next, 20 μl of F108-grafted DNA solution (either L1′ DNA, L2 DNA, or any combination of L1′ and L2 with 20 μl total volume), 35 μl washed 10% solid fraction colloids and 340 μl 1 × TE solution were combined. To swell the particles, 4 μl toluene was added into the tube, followed by 0.1–1 μl green or red BODIPY dye, depending on the particle species, A or B. The tube was then tightly sealed and wrapped with parafilm and slowly rotated overnight (not vortexed). To evaporate the toluene, the sample was settled by micro-centrifuge, opened and put into the pre-heated oven (80 °C ) for 20–40 min, with periodic mixing. To remove toluene and unreacted DNA, the particles were washed 4–6 times in 1 × TE solution to a total sample volume of 1 ml, as before. After the last wash, the supernatant was removed and 350 μl 1 × TE was added to adjust the volume fraction to 1%. DNA-grafted particles can be stored at 4 °C for at least 2 months.

### Crystallite formation

We prepared samples at three different size ratios (*σ*_A_/*σ*_B_=0.96±0.02, 0.88±0.05 or 0.85±0.05), by using three pair-wise combinations of three differently sized particles (diameters: 378±15, 392±8 and 445±25 nm). For each sample, the larger particle species was stained with Red BODIPY and considered ‘B', and the smaller stained with Green BODIPY and considered ‘A'. For each sample, the two types of DNA-grafted particles (200 μl total volume solution, each particle addition computed to yield 1:1 number stoichiometry; for example, for 392 and 445 nm particles, we add 81.2 μl 392 nm particles and 118.8 μl 445 nm particles, each at 1% solids volume fraction) were mixed in a 0.2 ml PCR tube and pelleted at 8,000 r.p.m. for 30 min. 194 μl supernatant was discarded leaving 6 μl of suspension. A volume of 1 μl 5-base linker (1,000 μM, see detailed structure above) and 3 μl NaCl solution in 1 × TE (1 M) were added to make total volume 10 μl and volume fraction of particles ∼20%. The pellet was mixed, and settled by micro-centrifuge. A large insulated cooler was filled with several liters of tap water heated to >45 °C. The sample was first melted in a small 50 °C bath, and mixed again. The PCR tube was then settled again by micro-centrifuge, wrapped tightly with parafilm and submerged completely in the larger hot water bath. The cooler lid was tightly closed and the quenching continued for ∼3 days. Once the cooler temperature was several degrees below the estimated crystal melting temperature, the samples were removed and quenched rapidly to room temperature. The crystallites in the PCR tube were gently pipetted into 200 μl 1 × TE buffer containing 300 mM NaCl.

### Crystallite ligation, mounting and confocal imaging

To permanently crosslink the crystallites prior to confocal microscopy, the DNA bridges between the particles are ligated, as described elsewhere^34^. The crystallites in 200 μl TE buffer were sedimented at 1 g overnight. The supernatant was removed totally and 300 mM NaCl in bio-water solution was added to bring the total volume to 30 μl. A volume of 4 μl ligase buffer and finally 4 μl ligase were added to the tube and allowed to react for 3 h at room temperature. After ligation, the 30 μl volume was diluted to a total volume of 200 μl with 300 mM salt solution. For mounting, 10 μl of ligated crystal suspension was placed onto a coverslip, the crystals allowed to sediment and bind for 10 min, followed by one drop of an high refractive index mounting solution. The mounting medium was then vacuum-dried overnight, and the sample sealed to a microscope slide with silicone vacuum grease.

The confocal microscope consisted of VisiTech confocal components, LEICA DM IRB optical microscope, with an Olympus × 100 oil lens. The software we used to take and analyse images was Voxcell, with the settings set as 512*512 imaging mode, 31 fps rate, and 30 Jump Average. The green channel imaging was processed with the 488 nm (80–90% intensity) excitation and illumination wavelength, 488 nm primary dichroic, 500LP barrier filter, 100 μm confocal aperture, detector gain as 40–50%, with a 14–17% offset. The red channel imaging was processed with the 561 nm (80–90% intensity) excitation and illumination wavelength, 568 nm primary dichroic, 580LP barrier filter, 100 μm confocal aperture, detector gain as 35–50%, with a 14–17% offset. The images were taken using a Z-capture series with a 0.3 μm nominal step size, and saved as tiffs. The saved images were viewed and analysed in ImageJ/FIJI.

### *In situ* crystallization

To understand crystal formation, we also crystallized samples on a DIC microscope (LEICA DMIRB) with a × 100 oil immersion objective and condenser, both of which were temperature controlled (BIOPTECHS). The particle sample was prepared as above, but at a total volume fraction of roughly 1%. The sample was well mixed and mounted in a sample chamber formed by a coverslip and slide separated with a silicone vacuum grease sealant. After mounting on the microscope, the temperature was gradually increased up to the melting temperature, *T*_m_, where particle aggregates broke apart. To form crystals, the temperature was quickly decreased to 1–2.5 °C below *T*_m_; crystals typically form in a few minutes and growth was completed in ∼30 min. To obtain larger crystals, after a few minutes of nucleation at the lower temperature, the temperature can be increased by 0.3–0.6 °C reducing the rate of further nucleation, and slowing the rate of crystal growth.

### Crystallographic determination of structure

We considered numerous binary structures and determined that they could not reproduce our observed crystallites. Some were easily rejected, as they were not members of the Cubic Crystal system suggested by our crystal faceting: Ag_2_Se, HrBr_2_, AlB_2_, AuTe_2_, γCuTi, CrB, MgZn_2_, MgNi_2_ and Wurtzite (ZnS). Within the Cubic System we closely examined the CsCl, NaCl, αIrV, Zindblende (ZnS), AuCu, Cu_3_Au, MgCu_2_, Cu_2_O, FeS_2_, ReO_3_, Cr_3_Si, Ag_2_O, CaF_2_ and Pt_3_O_4_ structures and found that none could reproduce our observations. None of the non-AB-type crystals displayed the same lattice in both colour channels. Of the AB-type cubic crystals all showed (100) facets with particle rows rotated 45° relative to those observed, significantly different interparticle spacing in their (100) and (111) planes or both. None of the crystals displayed any structures analogous to the (211) view of cubic diamond, along any viewing direction, except for the NaTl (or B32) lattice.

### Materials

The OptiLink Carboxylate-modified PS particles (405 nm nominal diameter, lot # 603850, 424 nm nominal diameter, lot # 300069, and 531 nm nominal diameter, lot # 903902) were purchased from Seradyn (now Thermo Scientific) and diameters found to be 378±15, 392±8 and 445±25 nm in diameter using Dynamic Light Scattering (DLS). Pluronic F108 Pastille was purchased from BASF Corporation. Dichloromethane (DCM, anhydrous, 99.8%), Triethylamine (TEA, 99%), 4-Nitrophenyl chloroformate (4-NPCF, 98%), and Touene (anhydrous, 99.8%) were purchased from Sigma-Aldrich. Tris-EDTA, 1 × (1 × TE, For Molecular Biology, pH=8.0) was purchased from Fisher BioReagents. Green dye (BODIPY, D3922) and red dye (BODIPY, D3835) were purchased from Invitrogen Company. T4 DNA Ligase (#M0202L) and T4 DNA Ligase Buffer (10 × , 10 mM ATP, #B0202S) were purchased from New England BioLabs. Bio-water (Biology Grade) was purchased from HyClone Company. Ethanol (200 Proof) was purchased from Decon Labs. Hydrochloric Acid (Certified A.C.S.) was purchased from Fisher Scientific. All the chemicals above were used as received. DNA strands (L1′, L2 and linker, see detailed structures above) were purchased from Integrated DNA Technologies (IDT) and diluted with bio-water as needed. Citric Acid (Certified A.C.S.), Sodium Carbonate (Certified, A.C.S.), Sodium Chloride (Certified, A.C.S.) were purchased from Fisher Scientific. The glass vials (3, 20 ml), were purchased from Fisher Scientific and washed before use. All Eppendorf tubes, PCR tubes, centrifuge tubes, and pipette tips were purchased from Fisher Scientific and were either pre-sterilized or autoclaved before use. The mount solution (IMMU-MOUNT, REF 9990402) was purchased from Thermo.

### Brownian dynamics simulations

Simulations were performed using the LAMMPS software package (http://lammps.sandia.gov/) with particle–particle interactions calculated using a coarse grained model reported earlier^24^. Large and small particles were assigned diameters of 445 and 392 nm, respectively, size ratio 0.88. Interactions between small particles were treated as purely repulsive, while large-large binding strengths ranged from 1 to 20 *k*_B_*T* and large–small binding strengths ranged from 5 to 30 *k*_B_*T*. The fluid viscosity was set to 10% that of water. The volume fraction of non-crystallized particles was initialized at 10%. Double-diamond crystallite seeds were initialized with sizes ranging from 50 to 4,000 particles in a cuboctahedral shape. Periodic boundary conditions were used for all simulations.

### Numerical evolution of zero modes

Zero frequency vibrational modes were identified by calculating the kernel of a crystals dynamical matrix. Any linear combination of eigenvectors within this kernel may then be chosen as a direction in which the lattice may be freely deformed. Once a direction, **r**_*n*_, is chosen from the kernel the system is displaced slightly in the direction of that mode. After this displacement, the dynamical matrix is recalculated and the kernel is searched for a new direction, **r**_*n*+1_, which maximizes **r**_*n*_˙**r**_*n*+1_. This process is continued until the dimensionality of the kernel reaches 6, indicating the only zero frequency modes remaining in the system are the six rigid translational and rotational modes.

### Order parameter for umbrella sampling simulations

The Steinhardt bond-orientational order parameter was used to identify crystalline particles^38^. We employed the basic strategy based on the **q**_6_**.q**_6_ measure suggested in ref. 36, modified slightly to accommodate the specifics of the DD configuration. Both A and B particles were considered in order parameter. In particular, we use a single cutoff distance for identifying neighbours that is 10% greater than the equilibrium B–B separation. For each particle with three or more neighbours, **q**_6_ is computed by averaging over the neighbours. **q**_6_ also is computed for each of the neighbour particles. Then **q**_6_˙**q**_6_ is computed for each neighbour pair; particles with at least three instances of a **q**_6_˙**q**_6_ above a threshold value are considered to be crystalline. Here the threshold value of **q**_6_˙**q**_6_ was set to be much lower for A–B and A–A pairs than for B–B and B–A pairs to accommodate the disorder associated with the A particles.

### Data availability

All original data sets produced as a part of this study are available from the corresponding author on reasonable request.

## Additional information

**How to cite this article:** Wang, Y. *et al*. Colloidal crystals with diamond symmetry at optical lengthscales. *Nat. Commun.* **8,** 14173 doi: 10.1038/ncomms14173 (2017).

**Publisher's note**: Springer Nature remains neutral with regard to jurisdictional claims in published maps and institutional affiliations.

## Supplementary Material

Supplementary Information Supplementary Figures 1-3 and Supplementary Table 1

Supplementary Movie 1 The movie shows a confocal focus (z) scan through two double diamond crystals having a cuboctahedral form. The image shows the smaller A spheres. The crystallite on the left rests on a (111) face, the one on the right rests on a (100) face. Z-resolution is not adequate to resolve separate crystal planes in depth, the observed image is a superposition of roughly 2 neighboring planes. The height of the image is 10.5 μm.

Supplementary Movie 2 The movie shows a confocal focus (z) scan through two double diamond crystals having that are irregularly faceted. The image shows the smaller A spheres. Both crystallites rest on a (211) facet, leading to the appearance of sphere doublets in a rectangular array. Z-resolution is not adequate to resolve separate crystal planes in depth, the observed image is a superposition of roughly 2 neighboring planes. The height of the image is 13 μm.
