# Supplementary material for: Colloidal crystals with diamond symmetry at optical lengthscales
Source: Nat Commun. 2017 Feb 13;8:14173. doi: 10.1038/ncomms14173 (PMC5316806; doi:10.1038/ncomms14173)
Supplement: Supplementary Information — Supplementary Figures 1-3 and Supplementary Table 1 [file ncomms14173-s1.pdf]

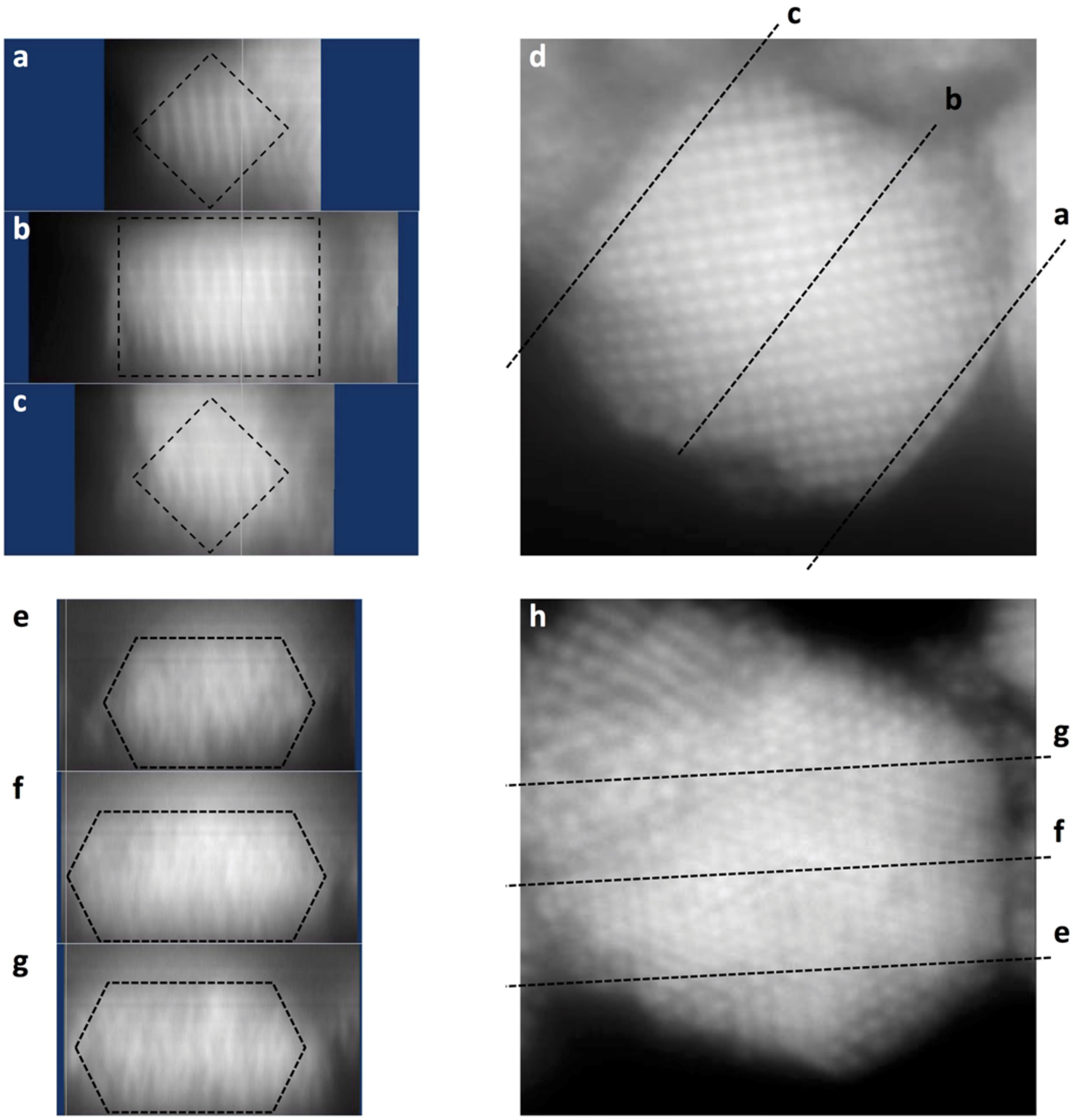

Supplementary Figure 1 | Confocal imaging reveals that crystallites are cuboctahedral. a-c, Show three vertical slices of a crystallite sitting on a 100 face, revealing the expected rhombic vertical faces at **a** and **c**, and a square cross-section at **b**. **d,** Shows the positions of the vertical slices on the corresponding ‘x-y’ image through the midplane. **e-g,** Show three vertical slices of a crystallite sitting on a 111 face, revealing the hexagonal cross-sections expected for a cuboctahedron. **h,** Shows the positions of the vertical slices on the corresponding ‘x-y’ image through the midplane. Crystallites correspond to those in Figure 2 in the main text.

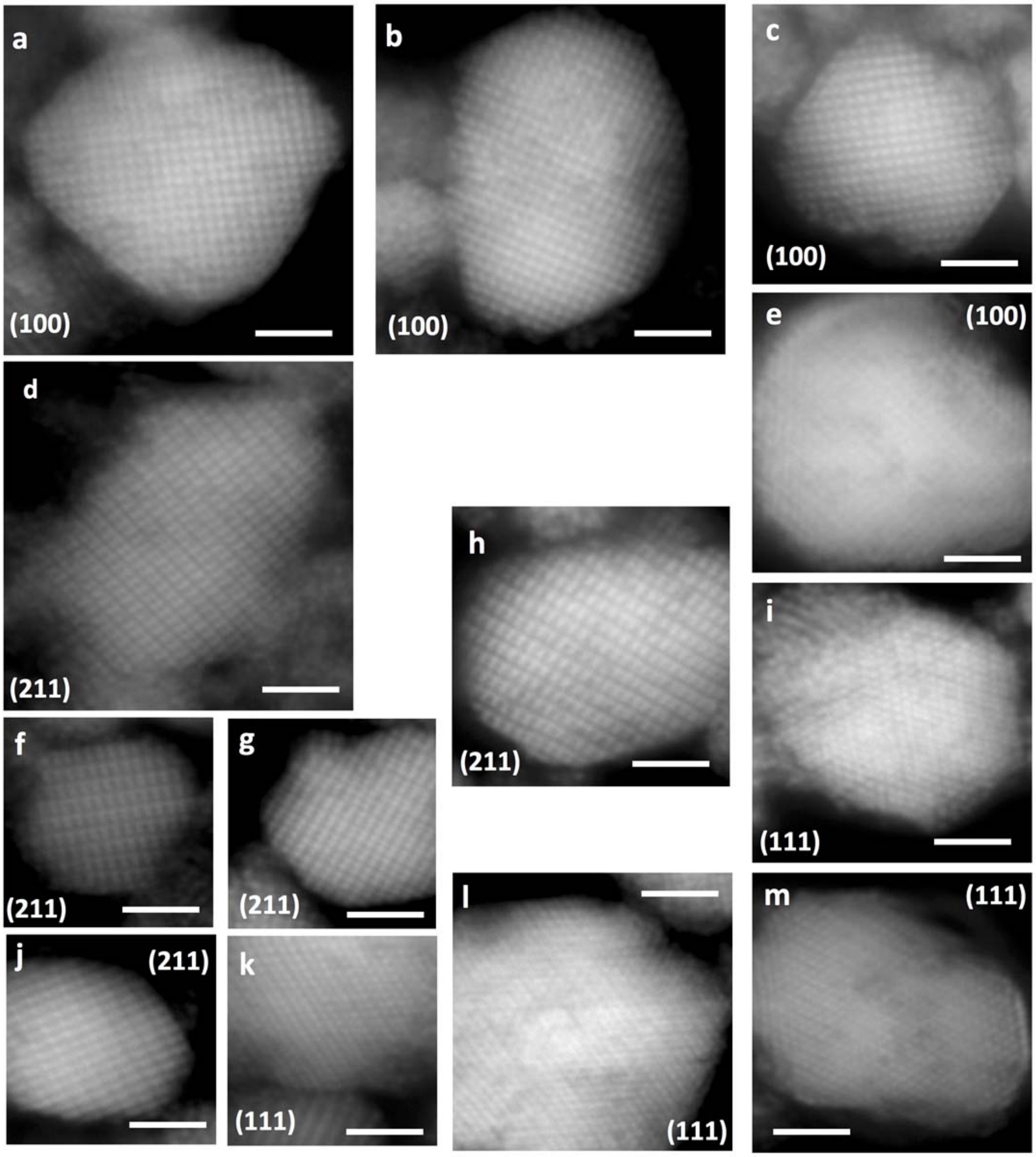

Supplementary Figure 2 | Particle size ratio 0.88 samples form DD crystallites. All confocal micrographs **a-m**, display unprocessed 2-dimensional slices in the midplane of 3-dimensional confocal scans of the crystallites and show the smaller 'A' particles (green fluorescence channel). The Miller index for the imaging plane of each crystallite is labeled. The depth resolution is greater than the size of a unit cell, multiple crystal planes are superimposed here. Images (**c,i**) and (**d,h**) are featured in the main text Figures 2 and 3 respectively. Experimental parameters for each image are summarized in Supplementary Table 1: (**a,b,c,d,f,g,h,i,j,m**) are condition 3, (**e,k,l**) are condition 4. All scale bars are 2 μm.

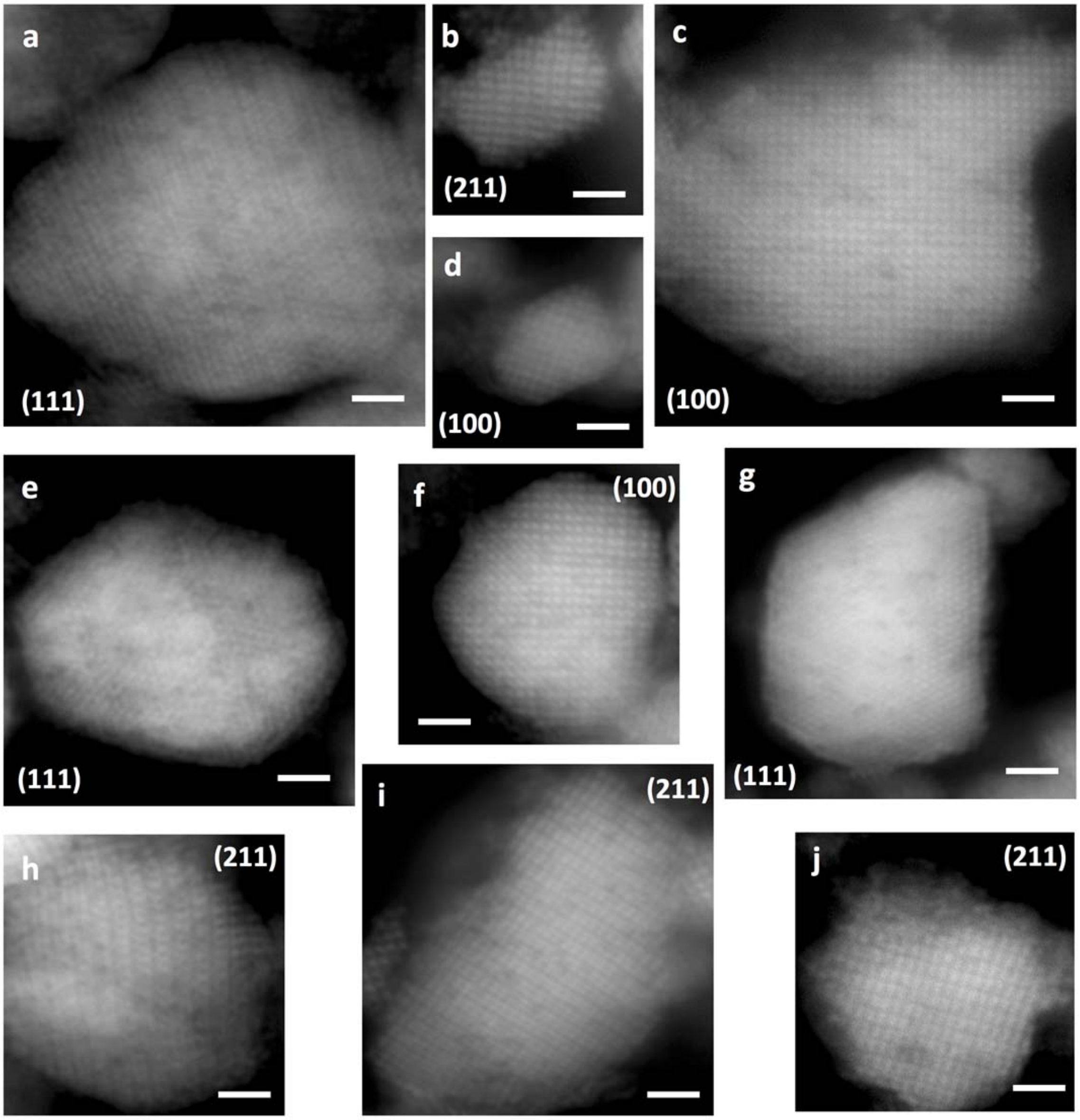

Supplementary Figure 3 | Particle size ratio 0.85 and 0.96 samples also form DD crystallites. All confocal micrographs **a-j**, display unprocessed 2-dimensional slices in the midplane of 3-dimensional confocal scans of the crystallites and show the smaller, 'A' particles (green fluorescence channel). The Miller index for the imaging plane of each crystallite is labeled. The depth resolution is greater than the size of a unit cell, multiple crystal planes are superimposed here. Experimental parameters for each image are summarized in Supplementary Table 1: (**b,c,j**) are condition 1, (**g**) is condition 2, (**e,h,i**) are condition 5, (**a,d,f**) are condition 6. All scale bars are 2 µm.

Double Diamond Crystallites Observed at Different Conditions						
Condition	1	2	3	4	5	6
Size Ratio (σ_A/σ_B) nm	0.85 (378/445)		0.88 (392/445)		0.96 (378/392)	
Mixing Ratio	α_A =0, α_B =0.1	α_A=0.1, α_B =0.1	α_A =0, α_B =0.1	α_A=0.1, α_B =0.1	α_A =0, α_B =0.1	α_A=0.1, α_B =0.1
Binding Strength	U_{aa}=0, U_{bb}>0	U_{aa}>0, U_{bb}>0	U_{aa}=0, U_{bb}>0	U_{aa}>0, U_{bb}>0	U_{aa}=0, U_{bb}>0	U_{aa}>0, U_{bb}>0
(100) face	1(0)	0	5(3)	3(2)	2(2)	3(1)
(111) face	4(2)	2(1)	8(5)	5(3)	4(2)	3(2)
(211) face	2(1)	1(1)	9(4)	4(2)	4(2)	1(1)
Total:	10		34		17	
DD% of All	10±3%		17±3%		10±3%	

Supplementary Table 1 | Double Diamond (DD) crystals were observed under six different conditions. Three different sized microspheres were taken pairwise, yielding three binary systems with different particle diameter (σ) ratios. For each size ratio, two different interaction matrices were considered, controlled by the mixing of complementary DNA strands on the smaller 'A' and larger 'B' particles. The mixing parameter α_X indicates the mole fraction of DNA strands on the 'X' particle that are complementary to the majority strand, that induce a 'like' attractive interaction between two 'X' particles whose strength is proportional to α_X. When the mixing parameter is zero, then the corresponding 'like' interaction is zero. The "(100) face" row, "(111) face" row, and "(211) face" row report the number of crystallites we observed in different orientations, while the numbers in the parentheses report the subset of those crystals that were slightly distorted as described in the text. The different experimental conditions were not uniformly sampled; more data was collected for some conditions than others. The final row provides an estimate of the fraction of crystallites in each sample that were DD, the remainder were CsCl-type. No DD crystals were observed (less than 3% incidence) for three other conditions (not shown) at these size ratios but with A-B type 'unlike' interactions only (α_A=0, α_B=0).
